# Artifact quantification and tractography from 3T MRI after placement of aneurysm clips in subarachnoid hemorrhage patients

**DOI:** 10.1186/1471-2342-11-19

**Published:** 2011-10-04

**Authors:** Faraz Khursheed, Fiona Rohlffs, Shuichi Suzuki, Dong H Kim, Timothy M Ellmore

**Affiliations:** 1 The Vivian L. Smith Department of Neurosurgery and Mischer Neuroscience Institute, The University of Texas Medical School at Houston, 6431 Fannin St., Houston TX 77030, USA; 2 Department of Diagnostic and Interventional Imaging, The University of Texas Medical School at Houston, 6431 Fannin St., Houston TX 77030, USA; 3 Neurointerventional/Neuro Endovascular, Radiological Sciences, University of California, Irvine, 101 The City Drive South, Rt. 140, South Bldg. 56, Mail Code: 5005, Orange, CA 92868, USA

**Keywords:** artifact, BOLD-fMRI, diffusion-weighted imaging, inferior fronto-occipital fasciculus, intracranial aneurysm clip, magnetic resonance imaging, subarachnoid hemorrhage, titanium alloy, uncinate fasciculus

## Abstract

**Background:**

The application of advanced 3T MRI imaging techniques to study recovery after subarachnoid hemorrhage (SAH) is complicated by the presence of image artifacts produced by implanted aneurysm clips. To characterize the effect of these artifacts on image quality, we sought to: 1) quantify extent of image artifact in SAH patients with implanted aneurysm clips across a range of MR sequences typically used in studies of volumetry, blood oxygen level dependent signal change (BOLD-fMRI), and diffusion-weighted imaging (DW-MRI) and 2) to explore the ability to reconstruct white matter pathways in these patients.

**Methods:**

T1- and T2-weighted structural, BOLD-fMRI, and DW-MRI scans were acquired at 3T in two patients with titanium alloy clips in ACOM and left ACA respectively. Intensity-based planimetric contouring was performed on aligned image volumes to define each artifact. Artifact volumes were quantified by artifact/clip length and artifact/brain volume ratios and analyzed by two-way (scan-by-rater) ANOVAs. Tractography pathways were reconstructed from DW-MRI at varying distances from the artifacts using deterministic methods.

**Results:**

Artifact volume varied by MR sequence for length (p = 0.007) and volume (p < 0.001) ratios: it was smallest for structural images, larger for DW-MRI acquisitions, and largest on fMRI images. Inter-rater reliability was high (r = 0.9626, p < 0.0001), and reconstruction of white matter connectivity characteristics increased with distance from the artifact border. In both patients, reconstructed white matter pathways of the uncinate fasciculus and inferior fronto-occipital fasciculus were clearly visible within 2 mm of the artifact border.

**Conclusions:**

Advanced 3T MR can successfully image brain tissue around implanted titanium aneurysm clips at different spatial ranges depending on sequence type. White matter pathways near clip artifacts can be reconstructed and visualized. These findings provide a reference for designing functional and structural neuroimaging studies of recovery in aSAH patients after clip placement.

## Background

Recent advances in aneurysmal subarachnoid hemorrhage (aSAH) management have led to consistent improvement in survival, with case fatality rates decreasing 0.9% every year without a rebound increase in the proportion of survivors with severe disabilities [1]. Most published data on aSAH use the Glasgow Outcome Scale (GOS) and modified Rankin Scale (mRS) that place patients with mild disabilities in a 'Good Recovery' category. However, even patients who make a good recovery based on these outcome scales suffer from varying degrees of cognitive deficits, including most prominently problems with memory [2,3]. These deficits impair day-to-day functioning, impact workplace productivity, and affect social interactions often during the most productive years of patients' lives. While a growing body of literature documents these cognitive deficits in aSAH survivors using objective batteries of neuropsychological tests [2-6], far fewer studies have attempted to relate cognitive deficits to specific neurobiological changes.

The acquisition of advanced neuroimaging sequences and application of recently developed analysis techniques promises to shed more light on the relationship between brain changes following aSAH and specific cognitive deficits. Such techniques include 1) high spatial resolution (1 mm^3^) structural MRI volumes to measure cortical thickness and gray/white matter density using voxel based morphometric techniques [7,8], 2) high temporal resolution (1-2 sec) blood oxygen level dependent functional MRI [9] to measure rest-state [10,11] and task-evoked hemodynamic changes [12], and 3) diffusion-weighted MRI (DW-MRI) to measure white matter integrity and connectivity [13-16]. A recent set of voxel-based morphometric studies employed structural MRI acquired at 1.5 tesla and demonstrated gray matter atrophy in multiple brain areas following aSAH that, in some cases, correlate with neuropsychological deficits [17-20]. However, many more systematic imaging studies will be required to unravel the complex set of brain changes caused by accumulation of subarachnoid blood, lesions induced by intraparenchymal hemotomas frequently seen with aneurysm rupture (Fisher Grade 4 [21]), vasospasm-induced ischemic damage, and surgical retraction/manipulation of local tissue. The widespread availability of high-field (3 tesla and greater) in clinical and research MRI centers will reduce the time to acquire advanced sequences and provide increased signal-to-noise ratios compared to lower field scanners [22-24].

Treatment of many aSAH patients involves the surgical placement of non-ferromagnetic (e.g., titanium alloy) intracranial aneurysm clips [25,26]. These patients are routinely scanned clinically at 1.5 tesla field strength [27,28] if the implanted clips are confirmed by written record to be made from titanium or other non-metallic materials that have been tested [29-37]. Clips made with ferromagnetic materials are unsafe even at low field strengths [38,39] and if operative records indicate these are implanted then patients cannot be scanned. Recent studies have assessed various characteristic (e.g., torque and translational attraction, MRI-related heating, and artifacts) in high-field strength MR environments of clips made from a range of non-ferromagnetic materials, including titanium alloy, at high field strengths from 3 up to 8 tesla [40-44]. While it may be routine clinical practice to obtain MRI scans in patients with these clips, the implants produce a noticeable artifact in the images. These artifacts have never been quantitatively described *in vivo *at high field (> 1.5 tesla) in aSAH patients, although basic characteristics of artifacts have been reported for imaging obtained with clips in phantoms at 1.5 tesla and higher [29,34,45,46].

Given previous studies of MRI artifacts from aneurysm clip implants in humans conducted at low field strength and studies at both low and high field strengths conducted with phantoms, we expected at the outset of this study to find that artifact size depends on the type of pulse sequence. The novelty of our study is that it is conducted at 3T *in vivo *with patients who have a history of SAH, and that artifact size is quantified across a range of acquisitions using parameters that are typically found in advanced research MRI studies of cognitive function. The major motivation to characterizing the MR artifact *in vivo *rather than in phantoms is that the results can be interpreted from a clinically-relevant perspective in order to give neurosurgeons, neuroradiologists and researchers alike an idea of how much signal loss varies across these acquisitions before they embark on large-sample imaging studies of neurocognitive recovery after SAH. A description of artifact characteristics obtained *in vivo *across a range of advanced image acquisition sequences is necessary in order to understand the extent of signal integrity around the aneurysm clip. Only after the boundary of signal loss around the clip is quantified can the signal changes arising from damage due to the hemorrhage, normal signal change from healthy brain tissue, and connectional neuroanatomy near implanted clips, like that made possible by DW-MRI tractography, be adequately summarized.

### Objectives

Image artifact due to the presence of titanium alloy aneurysm clips is generally acknowledged to be minimal. Yet no *in vivo *data is available from aSAH patients to guide clinicians and researchers in estimating the extent of aneurysm clip artifact in images reconstructed from advanced high-field (> 1.5 tesla) MR sequences. In this paper, we test the null hypothesis that the resulting artifact from implanted titanium alloy clips is spatially similar across high-resolution T1- and T2-weighted structural, T2* echo-planar BOLD-fMRI, and DW-MRI images when clinical characteristics related to the extent of aSAH and clip characteristics are similar. We also explore the ability to reconstruct temporal and frontal lobe white matter fiber pathways reconstructed using deterministic tractography methods from the DW-MRI acquisitions, and quantify pathway density at different distances from the artifact border.

## Methods

### Patients

Two patients with a history of SAH who were treated by placement of titanium alloy aneurysm clips were imaged after giving informed verbal and written consent for enrollment in the study. Patients gave informed consent for their brain images to be published. This research study was approved by The Committee for the Protection of Human Subjects of The University of Texas Health Science Center at Houston (HSC-MS-09-0497). The two patients were similar in terms of the degree and distribution of SAH, its intraparenchymal component, the degree and severity of vasospasm [47], the surgical approach taken, and characteristics of implanted clips, including composition, *in-vivo *clip orientation, and dimensions (for details of each, see Table 1). We did not study patients with clips made from Phynox, MP35N, and Elgiloy, as these clips are not routinely implanted at our institution, nor did we study patients with curved, right-angle or other more complicated clip configurations.

**Table 1:** Patient Demographics and Aneurysm Clip Characteristics

	*Patient A*	*Patient B*
***Patient Demographics***		

*Age*	62	36

*Sex*	F	F

*Hunt and Hess Grade*	3	4

*Fisher's Grade*	4	4

*SAH Distribution*	Bilateral, L > R	Bilateral, L > R

*Aneurysm location*	ACOM	Left ACA

*Intraparenchymal Component*	Left Frontal	Left Frontal

*Focal Neurological Deficits*	Yes	Yes

*Vasospasm*	Clinically significant requiring endovascular intervention	Clinically significant requiring endovascular intervention

		

***Surgery ***		

*Approach*	Left Pterional	Left Pterional

*Operative findings*	Hemosiderin deposition on brain surface	Significant subarachnoid blood

*Time between SAH and Surgery*	11 months	9 days

*Time between Surgery and 3T MR Scan*	5 months	15 months

		

***Clip Specifications***		

*Manufacturer*	Aesculap Yasargil FT720T (Aesculap, Germany)	Aesculap Yasargil FT760T (Aesculap, Germany)

*Composition *	Titanium Alloy Ti6A14V ISO 5823-3	Titanium Alloy Ti6A14V ISO 5823-3

*Size*	7 mm (mini)	11 mm (Standard)

*Weight*	0.08 g	0.19 g

*Width*	0.95 mm	1.35 mm

*Shape*	Straight	Straight

### Image Acquisition

The following brain scans were obtained using a 3T Philips Intera scanner (Philips Medical Systems, Bothell WA; 8-channel SENSE acquisition): (1) T2-weighted turbo-spin echo 3D volume acquisition (repetition [TR]/echo time [TE] = 2500/367 ms; ETL 120, pixel bandwidth 380, FA = 90 degrees, matrix size = 256 × 256; field of view [FOV] = 240 mm, 186 0.94 mm thick contiguous, sagittal slices), (2) high-resolution 3D T1-weighted magnetization-prepared rapid acquisition turbo field echo sequence (TR/TE = 8.4/3.9ms; FA = 8 deg; matrix size = 256 × 256; FOV = 240 mm; slice thickness = 1.0 mm thick sagittal slices), (3) a set of diffusion-weighted image volumes (32-directions high angular resolution) using the gradient overplus option with one B0 (non-diffusion weighted) image volume acquired before the acquisition of one repetition of the diffusion-weighted scans (TR/TE = 8500/67 ms; FA = 90 deg; matrix size 128 × 128; FOV = 224 mm; 2 mm thick axial slices, b-value of 800 s/mm^2^), (4) a set of functional images obtained with a gradient-recalled echo-planar imaging (EPI) sequence sensitive to the blood-oxygen level dependent (BOLD) signal (TR/TE = 2000/30 ms; FA = 90 deg; matrix size = 80 × 80; FOV = 220 mm; 3 mm thick axial slices, 150 dynamics). These particular sequence parameters were selected for use here because they are commonly applied in structural and functional studies of cognition in both healthy controls [48] and different patient groups [49,50]. Identical MR parameters were used to scan both patients; no attempt was made to adjust these parameters on an individual patient basis to minimize artifact extent.

### Image Analysis

Image visualization and artifact quantification was performed using the AFNI package [51]. Two raters with fellowship training in neuroimage analysis and who were blind to each other's results performed intensity-based planimetric contouring to define each artifact. The T2, EPI and DW image volumes were aligned to the T1 volume using the 3dAlineate alignment algorithm with a mutual information cost function in AFNI. Each rater created 3D binary masks on the aligned volumes that encompassed the entire spatial extent of the artifact on each patient's T1, T2, mean EPI, and the B0 diffusion-weighted scans. The artifact was clearly visible in each of the four different acquisitions, and verified numerically to consist of a low intensity (i.e., black) region wherein the highest intensity image value at the center of the artifact was verified across all patients and all scans to never exceeded 10% of the global image maximum. The consistent behavior of the artifact intensity allowed for the choice of an intensity-based threshold to be automatically chosen during 3D contouring of the artifact border. After the artifact was delineated, the long-axis and total volume for each of these binary masks was then measured by summing mask voxel volumes. Most importantly, for comparison across patients and MR sequences, artifact volume was expressed as a percentage of whole brain volume, defined as the total gray and white matter of the brain as computed in Freesurfer v4.5 [52,53].

To determine whether the clip artifact differed significantly as a function of scan sequence and rater, two 2-way analyses of variance were conducted using SPSS (v.13). In the first ANVOA, the dependent variable was the artifact length/clip length ratio, and the two fixed factors were rater (2-levels: rater 1 & 2) and scan sequence (4-levels: T1, T2, EPI, and DW). In the second ANOVA, the dependent variable was the artifact volume/brain volume ratio, and the two fixed factors were rater (2-levels: rater 1 & 2) and scan sequence (4-levels: T1, T2, EPI, and DW). These ANOVAs had 3 and 15 numerator and denominator degrees of freedom, allowing for *a priori *p < 0.05 statistical threshold for the fixed effects of rater and scan sequence. Due to the few patients studied, no statistical interpretation about the random effect of patient was possible.

To quantify how the number of white matter tractography pathways varied as a function of distance from the artifact, diffusion tractography pathways were computed from the set of aligned diffusion-weighted images according to previously described methods [54,55]. Then, the artifact mask delineated on the diffusion-weighted scan was morphologically dilated in three dimensions by increments of the native-space DW voxel resolution (2 mm), and all pathways intersecting with the dilated mask were saved and visualized in DTI Query [56].

## Results

The means and standard deviations of the two raters' measurements of each patient's artifact are listed by imaging sequence in Table 2.

**Table 2:** Artifact Measurements as a Function of MR Sequence

	Sequence	Artifact Volume mm^3^	% Of Brain Volume	Artifact Length mm	Artifact/Clip Length Ratio
**Patient A**	*T1*	1038 (366)	0.45 (0.16)	25.31 (0.09)	3.61

*(7 mm clip)*	*T2*	484 (185)	0.21 (0.08)	20.28 (1.80)	2.89

	*EPI*	7366 (4767)	3.21 (2.08)	45.91 (4.72)	6.55

	*DW*	3792 (168)	1.65 (0.07)	36.62 (2.39)	5.23

					

**Patient B**	*T1*	2223 (428)	0.92 (0.18)	34.52 (1.19)	3.18

*(11 mm clip)*	*T2*	1093 (101)	0.45 (0.04)	31.05 (0.21)	2.82

	*EPI*	9557 (2728)	3.94 (1.13)	52.33 (4.41)	4.75

	*DW*	6897 (2644)	2.85 (1.09)	42.97 (0.36)	3.90

The mean of the two raters' estimates is listed with standard deviation in parentheses.

For both patients, the artifact appeared smallest on T2-weighted structural volume (0.21% to 0.45% of whole brain volume, artifact/clip length ratio of 2.82 to 2.89) and the largest on the EPI functional image (3.21% to 3.94% of whole brain volume, artifact/clip length ratio of 4.75 to 6.55).

Significant main effects of scan type were found by the ANOVA of artifact length/clip length ratio (F_(3,15) _= 8.51, p = 0.007), and the ANOVA of artifact volume/brain volume ratio (F_(3,15) _= 22.25, p < 0.001) supporting the hypothesis the artifact size varies among the different MR acquisition sequences. Further evidence was found by computing post-hoc tests for significant (p < 0.05) differences among the means (Bonferroni corrected for multiple comparisons). These showed differences between the structural (T1 and T2) and EPI sequences for the artifact length/clip length ratios, and between the structural (T1 and T2) and EPI and DW sequences for the artifact volume/brain volume ratios. Differences between the EPI and DW artifact volume/brain volume ratios trended toward but failed to reach significance (p = 0.11).

Inter-rater reliability for all measurements (artifact volume, artifact as percentage of brain volume, and artifact length) was very high (r = 0.9626, p < 0.0001) demonstrating objective differentiation between raters of the artifact size as a function of MR sequence. In general, inter-rater variability in estimation of artifact length was low (Range: 0.21-1.80 mm for T2, 4.41-4.72 mm for EPI) and artifact volume was relatively high (Range: 101-185 mm^3 ^for T2, 2728-4767 mm^3 ^for EPI). There were no significant main effects of rater found in either the ANOVA of artifact length/clip length ratio or the ANOVA of artifact volume/brain volume ratio, showing that estimates of artifact size were consistent and did not differ systematically by individual rater.

**Figure 1:**
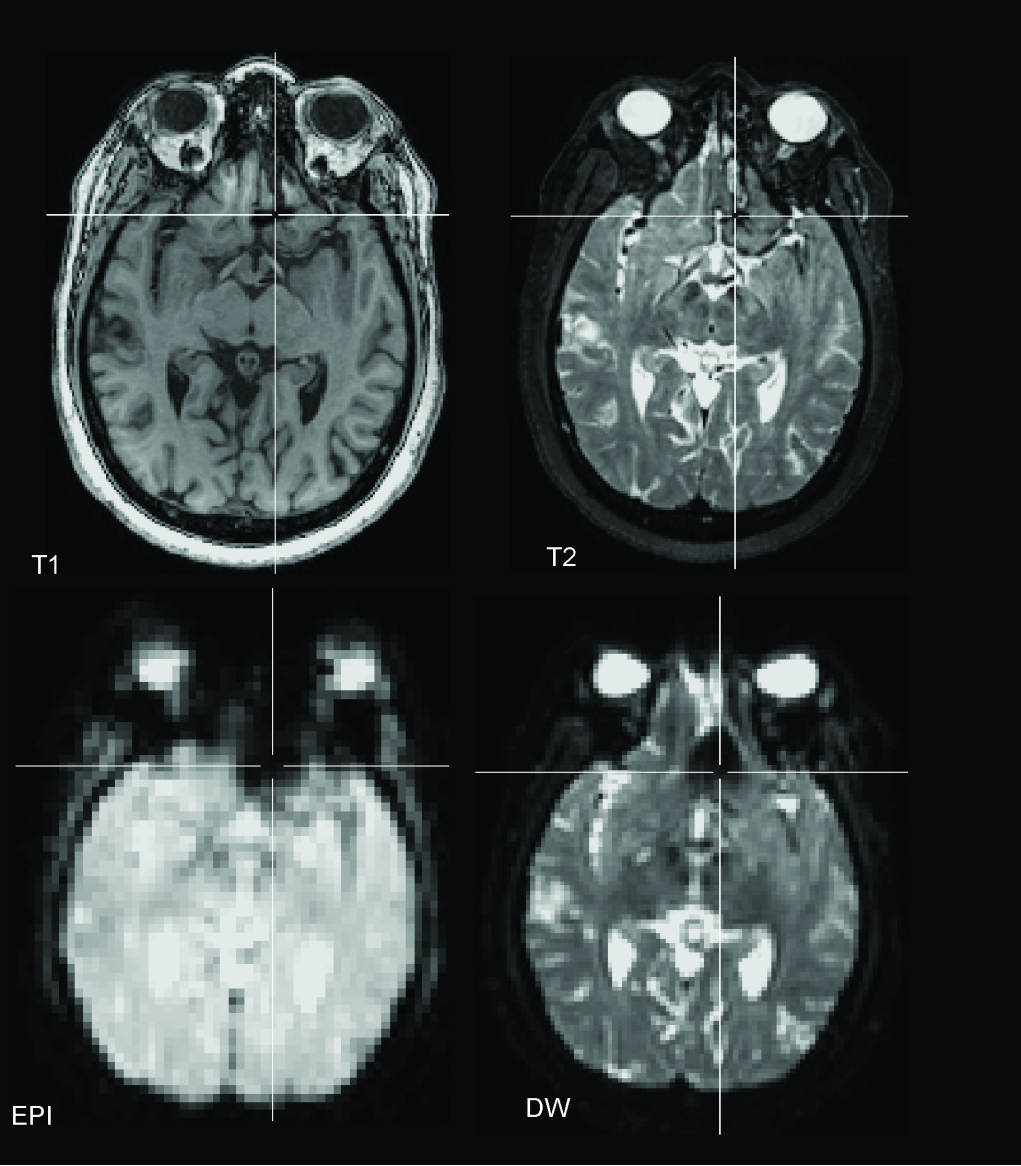
**Appearance of Aneurysm Clip Artifact on Different Aligned MRI Sequences**. Axial slices taken in the same imaging plane for Patient A are displayed in the axial plane (radiological convention) with the crosshair placed in the center of the clip artifact.

Artifact size differences are easily visualized by comparing an image plane across different MR sequences taken through the center of the artifact (Figure 1). On all sequences in both patients, the maximum length measured along the long axis of the artifact was larger than the actual clip length, ranging from 2.82 times the length of the clip for the T2 structural sequence to 6.5 times the length of the clip for the EPI sequence.

**Figure 2:**
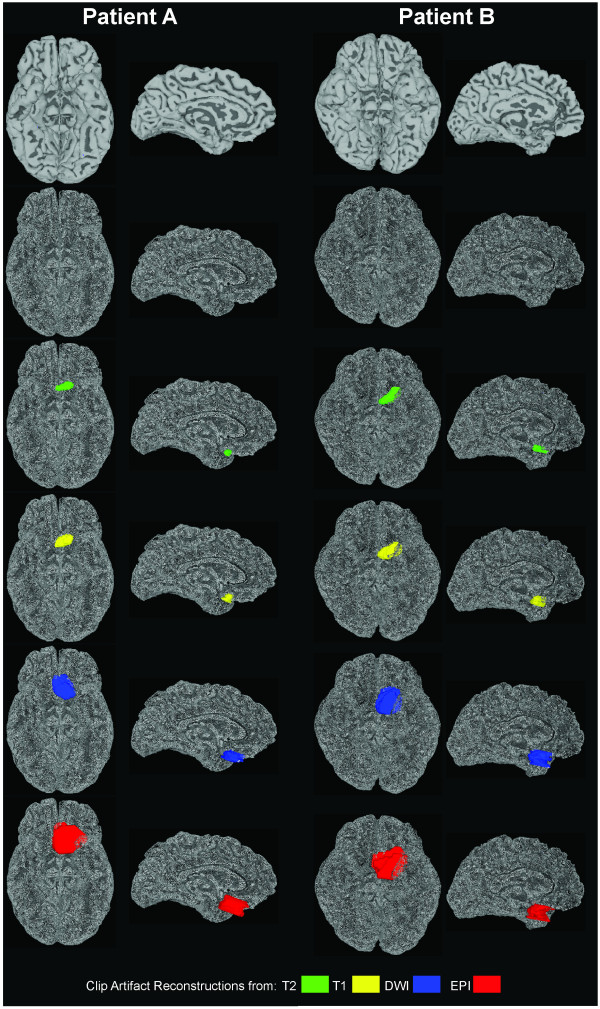
**Example Clip Artifact Volume Reconstructions in Relation to Cortical Surface Models**. Basal and mid-sagittal views of each patient's cortical surface model are shown shaded by curvature (top row) and as a point cloud (second row). Superimposed on the point could displays to aide artifact visualization are the reconstructed clip artifact volumes colored according to MR sequence in descending order by clip volume as percentage of brain volume (T2 = green, T1 = yellow, DWI = blue, EPI = red). These artifact volumes are 0.27%, 0.56%, 1.70%, and 4.67% respectively for Patient A (two columns on left), and 0.48%, 1.04%, 3.62%, and 4.74% respectively for Patient B (two columns on right).

Computed whole-brain volumes were 229,705 and 242,419 mm^3 ^for patient's A and B respectively. These values are in line with an average of whole brain volumes, 235,821 (SD 22,571), computed from a group of similar aged controls (N = 7, 3F, 1LH, 54.0 years, SD 7.8) published recently [49]. To help aide visualization of clip artifact volume in relation to brain size, example artifact volume reconstructions from the range of MR sequences are shown in relation to patient cortical surface models in Figure 2.

**Figure 3:**
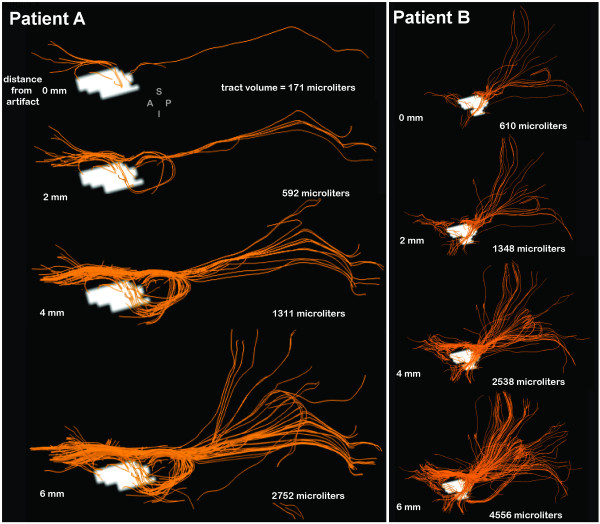
**White Matter Tractography Reconstruction Ability Increases as a Function of Distance from Artifact Border**. The artifact masks derived from the diffusion-weighted image volume were dilated in 2 mm increments, and the volume of white matter tracts that intersected any component of the dilated mask was computed. Components of the uncinate fasciculus (loop from temporal to ventral frontal) and inferior fronto-occipital fasciculus (occipital to temporal) in both patients' left hemisphere can be resolved within 2 mm of the artifact border by visual inspection of the orange tractography pathways. Both views are sagittal left hemisphere where A, P, S, and I indicate anterior, posterior, superior and inferior directions.

The ability to reconstruct nearby white matter fiber pathways as a function of distance from the artifact border was examined in both patients. The artifact mask delineated on the diffusion-weighted sequence was expanded by 3D morphological dilation incrementally in 2 mm steps (Figure 3) and the volume of the white matter tracts intersecting the dilated mask was found to increase markedly from the border, from 592 micro liters within 2 mm of the artifact border to 2752 micro liters within 6 mm of the border for Patient A with the 7 mm clip, and from 1348 to 4556 in Patient B with the larger 11 mm clip. After just one increment of dilation, pathways of the uncinate fasciculus (connecting anterior temporal and ventral frontal lobe) and pathways of the inferior fronto-occipital fasciculus, connecting parieto-occipital and ventral frontal lobe [57-59], were visible in the data of both patients (Figure 3).

## Discussion

Widespread application of advanced imaging techniques to investigate changes in brain structure, function, and connectivity following SAH is presently slowed by the fact that interpretation of the images obtained from patients with implanted aneurysm clips is complicated by the presence of an artifact in and around the clip. The spatial extent of the artifact with respect to clip size and type of MR sequence has not been previously described from images collected from aSAH patients *in vivo*. The main objective in conducting this study was to describe the extent of image artifact produced by implanted titanium aneurysm clips in aSAH patients across a set of advanced MR sequences necessary to quantify structural, functional, and connectivity patterns. A secondary objective was to describe using DW-MRI tractography methods how well white matter connectional patterns could be reconstructed around the clip artifact. This second objective is critical, we believe, for beginning to understanding how well modern neuroimaging approaches to studying white matter can be applied in SAH patients who have implanted aneurysm clips.

We focused on just two subarachnoid hemorrhage patients with similar clinical characteristics, including surgical approach, aneurysm location, Fisher's SAH grade [21], global distribution of subarachnoid blood, and implanted clip size and orientation. We acknowledge several limitations of this approach. First, the clinical characteristic of these patients were not matched exactly. Second, the small number of patients studied does not allow generalization of the findings to the entire population of aSAH patients who are treated neurosurgically with clips. We cannot predict for certain based on the findings presented here how artifact will manifest in patients with clips placed on, for example, the middle cerebral or basilar arteries. However, the two patients imaged in this study had clips placed after rupture of ACOM and ACA. Estimates based on large sample studies of western [60] and eastern [61,62] patient populations indicate that these locations account for between 29 to 38 percent of total intracranial aneurysm ruptures. This suggests that, despite the limited scope of our study, our findings may be generalizable to a sizeable subset of the population with titanium clips placed at these locations. Finally, we studied patients implanted only with straight titanium alloy clips. Patients with more complex clip configurations might demonstrate a larger artifact relative to a straight clip of same size and weight. Furthermore, more SAH blood distribution around the clip, more aggressive surgical manipulation of surrounding tissue and more complex clip configuration would likely increase artifact size relative to the estimates presented here. In our limited study, two patients studied were scanned at 3 tesla, and high resolution T1- and T2-weighted structural, BOLD-fMRI, and DW-MRI sequences were acquired. The image volumes were aligned to the native image space of the high resolution T1 anatomical scan, and experienced raters used semi-automated tracing methods to delineate the image artifact around the aneurysm clip on each set of image volumes. For each sequence, a 3D mask of the artifact was produced. Mask volumes were compared across sequences and between raters. Inter-rater reliability was highly significant, indicating that the extent of the image artifact was reproducible across sequences. Most importantly, we found evidence to reject our initial null hypothesis that the spatial extent of the image artifact is the same across 3T MR acquisition sequences. Our main result is that the size of the clip artifact varies considerably; it is smallest in volume on the structural scans and largest on the echo-planar BOLD-fMRI scans, with the artifact on the DW-MRI sequence falling in between.

The result from our second objective is that we were surprised to learn that it is possible to reconstruct white matter pathways very close to the artifact border using DW-MRI data. For the two patients studied here, pathways comprising uncinate and inferior fronto-occipital fasciculi [57-59] were both visible within 2 mm of the artifact border. After applying a three dimensional artifact mask generation method, we also provide multiple objective measures, including an artifact/clip-length ratio and artifact volume/brain tissue volume ratio that can be used to guide neuroimaging researchers in estimating the size of the artifact if clip characteristics like length are known.

In hindsight, it is not too surprising that the clip artifact differs considerably in size given that the sequences required to measure structural, functional, and diffusion-weighted connectivity information differ according to radiofrequency pulse design, gradient readout times, and spatial resolution. It is necessary that these parameters differ in order for scanners to be capable of measuring different MR signal characteristics, and it is important to know how clip artifact size will differ as a function of type of imaging sequence. In this study, we made no attempt to adjust parameters in individual patients to minimize artifact. This was a deliberate choice made to minimize signal variance as a function of acquisition parameters so that within-group (SAH) analyses and between-group (SAH vs. matched control) can be conducted in future larger-sample studies. For functional imaging, there is a necessary tradeoff between high spatial and temporal resolution when acquiring fMRI-BOLD sequences. In our echo-planar fMRI sequence, we used a 2 second repetition time, meaning that hemodynamic activity throughout 34 slices representing the entire volume of the brain is captured every two seconds. This acquisition speed is common for task-evoked and rest state designs. Fast fMRI acquisitions require larger voxel sizes of ~ 3 mm in order to image most of the brain. Advances in head coil design, including the number of channels to record the signal, will in the future allow acquisition of higher spatial resolution scans in a shorter time, but the spatial-temporal tradeoff will remain.

Artifact size in the fMRI-BOLD sequence acquired here is also significantly magnified by a susceptibility artifact at the base of the brain, near where many aneurysm clips are likely to be placed. This well-known dropout of signal due to a large static magnetic field inhomogeneity that occurs near air/brain interfaces is also present in echo-planar acquisitions in healthy controls. The interaction between clip location and signal dropout due to the brain tissue/air cavity susceptibility complicated delineation of the clip artifact because both artifacts appeared as low intensity regions and overlapped to some extent. However, the tissue-air artifact extends outside the border of the brain, so by constraining our artifact delineation to within-brain borders as defined by the high-resolution anatomical images we were able to minimize this error to some extent.

Outside the boundary of our artifact masks, imaging signal integrity recovers quickly. This was demonstrated by reconstructing diffusion tensor tractography fiber pathways as the artifact mask volumes were parametrically dilated in three dimensions. As we used standard diffusion tensor imaging methods here with relatively few gradient orientations, future investigations should evaluate the feasibility of collecting higher direction diffusion-weighted sequences in order to more accurately reconstruct white matter pathways in SAH patients using methods like Q-ball [63] and diffusion spectrum techniques [64]. Nevertheless, during dilation, the mask volume was expanded to include tissue outside the artifact and the number of tractography pathways increased rapidly as the mask was dilated at successively higher iterations, indicating an increase in our ability to examine connectivity characteristics just a few millimeters away from the artifact mask. This is critically important as aSAH may involve subtle damage to brain tissue near the rupture site. The damage may cause atrophy of nearby gray matter, altered connectivity of nearby white matter to other brain areas, or a combination of both. Altered network connectivity between brain regions near the rupture site (i.e., hippocampal complex) and regions distal to the rupture site (i.e., ventrolateral prefrontal cortex) is one hypothesis that could account for complex deficits [4], like those of memory, mood, and decision making, that have been reported in aSAH patients. To adequately test this hypothesis, white matter connectivity, for tracts thought to be important for memory like the uncinate fasciculus (shown in Figure 3 for both patients) must be reconstructed, and the extent of artifact due to the aneurysm clip must be quantifiable and reproducible in order to know in which areas connectivity information is reliable. The results presented here show how this may be achieved through construction of a spatial confidence boundary of signal integrity, represented numerically by an artifact mask volume. These volumes should be constructed from the MR imaging data obtained from each aSAH patient. Across patients, the spatial extent of these artifact masks may be considered in a group analysis of the imaging data. Using this approach, it would be possible to quantify the degree of uncertainty across patients about whether signal in a particular region of the brain is completely or partially affected by clip artifact. Importantly, this method is also generalizable to other methods of assessing connectivity, like functional connectivity computed from rest-state fMRI [65].

## Conclusions

In summary, we show that artifact due to the presence of titanium clips differs across a range of advanced high-field (3 tesla) MR imaging sequences in aneurysmal subarachnoid hemorrhage patients. This artifact is easily quantifiable and 3D volumes of confidence can be produced outside of which the MR signal for measuring structure, function, and diffusion-weighted connectivity information may be obtained. We also show that DW-MRI tractography can reveal white matter pathways in close proximity to the artifact borders. These results have important methodological implications for studying brain changes with advanced neuroimaging tools in patients who recover from subarachnoid hemorrhage after neurosurgical treatment.

## Funding

This project was supported by the Brain Aneurysm Foundation and the Vivian L. Smith Foundation for Neurological Research. Partial funding for the purchase of the Philips 3T scanner used to collect the imaging data was provided by NIH S10 RR19186. No funding was provided by manufacturers of clips studied.

## Abbreviations

3T: three tesla; AFNI: Analysis of Functional Neuroimages; ANOVA: analysis of variance; ACA: anterior cerebral artery; ACOM: anterior communicating artery; aSAH: aneurysmal subarachnoid hemorrhage; BOLD: blood oxygen level dependent; DTI: diffusion tensor imaging; DW-MRI: diffusion-weighted magnetic resonance imaging; EPI: echo-planar imaging; ETL: echo train length; FA: flip angle; fMRI: functional magnetic resonance imaging; FOV: field of view; GOS: Glasgow Outcome Scale; mRS: modified Rankin Scale; MRI: magnetic resonance imaging; TE: echo time; TR: repetition time

## Competing interests

The authors declare that they have no competing interests.

## Authors' contributions

FK, FR, and TME collected the MRI data. DK did the surgeries and referred patients for research. FK and TME analyzed the data. SS helped interpret the imaging data. FK and TME wrote the paper. All authors read and approved the final manuscript.

## Pre-publication history

The pre-publication history for this paper can be accessed here:

http://www.biomedcentral.com/1471-2342/11/19/prepub
